# Benefits of different characteristics of pulmonary rehabilitation programs in patients with interstitial lung disease: a systematic review and meta-analysis

**DOI:** 10.1080/07853890.2025.2566868

**Published:** 2025-10-27

**Authors:** Qian Chen, Yifan Qiu, Mengchun Xu, Liang Dong

**Affiliations:** ^a^ Department of Respiratory, Shandong Qianfoshan Hospital, Cheeloo College of Medicine, Shandong University, Jinan, China; ^b^ Department of Respiratory, The First Affiliated Hospital of Shandong First Medical University and Shandong Provincial Qianfoshan Hospital, Shandong Institute of Respiratory Diseases, Featured Laboratory of Respiratory Immunology and Regenerative Medicine in Universities of Shandong, Jinan Clinical Research Center for Respiratory Disease, Jinan, China

**Keywords:** 6-min walk test, meta-analysis, interstitial lung disease, pulmonary rehabilitation, systematic review

## Abstract

**Background:**

Interstitial lung disease (ILD) is defined by progressive pulmonary fibrosis, restrictive ventilatory dysfunction, and hypoxemia, with significant differences in disease presentation, clinical course, and prognosis. This study aims to evaluate the clinical benefits of pulmonary rehabilitation with different characteristics in ILD patients.

**Methods:**

This study searched electronic databases (PubMed, Embase, Web of Science, Cochrane Library, Clinical trials) and included 33 articles in a meta-analysis (1671 ILD patients). Two investigators independently appraised relevance, trial quality, and data extraction.

**Results:**

The meta-analysis demonstrated that pulmonary rehabilitation is effective in enhancing exercise endurance, alleviating exertional hypoxemia, and improving quality of life among ILD patients. Key outcomes (all *p* < 0.05): 6-min walk distance (6MWD) [MD = −46.04; 95% CI (−52.63, −39.45)], lowest oxygen saturation during the 6-min walk test (6MWT) [MD = −1.97; 95% CI (−2.11, −1.84)], Borg scale score [MD = 1.20; 95% CI (0.79, 1.60)], and the MOS 36-item Short Form Health Survey (SF-36) [MD = −4.57; 95% CI (−7.19, −1.94)]. Subgroup analyses were performed based on duration, content, and implementation methods. Patients with 4–8 weeks duration showed optimal benefits. Content including breathing training improved exercise endurance more effectively. Medical guidance, home-based, and tele-rehabilitation all enhanced endurance, with no significant differences.

**Conclusion:**

Pulmonary rehabilitation has a positive therapeutic effect on ILD patients, significantly enhancing exercise endurance and tolerance, and improving quality of life. However, it is essential to assess the patient’s condition and tailor the pulmonary rehabilitation plan to the individual to achieve the best therapeutic outcomes.

## 1. Introduction

Interstitial lung disease (ILD) denotes a heterogeneous array of disorders affecting the alveoli and lung interstitium. Pathologically, ILD is defined by chronic inflammation or fibrotic remodeling of these structures, whereas clinically, it manifests as a progressive decline in pulmonary function, impaired diffusion capacity, and ultimately, respiratory failure and mortality [1]. Its marked heterogeneity makes early diagnosis and treatment challenging [2], thus positioning pulmonary rehabilitation as a crucial component in its management.

The 2013 ATS/ERS report on key concepts and recent advancements in pulmonary rehabilitation concludes that pulmonary rehabilitation represents a comprehensive multidisciplinary intervention framework, anchored in thorough individual assessment. It combines exercise training with individualized programs such as daily behavior education, aiming to alleviate symptoms of dyspnea, enhance exercise endurance, and boost quality of life [3].

Originally, pulmonary rehabilitation was implemented in the management of patients with chronic obstructive pulmonary disease (COPD) and has since been progressively extended to other chronic respiratory diseases [4]. Despite potential pathophysiological differences, ILD patients often exhibit symptoms similar to COPD patients. Thus, multiple guidelines from the American Thoracic Society, European Respiratory Society, and Australian and New Zealand Respiratory Society recommend pulmonary rehabilitation as a core non-pharmacological intervention for ILD [5–7]. The irreversible loss of lung function often manifests as the progression of interstitial lung disease. Notably, Studies have shown [8,9] that regular pulmonary rehabilitation exercise therapy can alleviate symptoms of dyspnea and improve the 6-min walk distance (6MWD).

The 6-min walk test (6MWT) quantitatively evaluates the integrated cardiorespiratory exercise function in patients with moderate to severe lung diseases by simulating daily activity energy expenditure patterns [10]. In comparison to cardiopulmonary exercise testing, this assessment demands no sophisticated equipment or specialized skills, making it more patient-friendly. As an assessment tool, the 6MWT demonstrates robust reliability, validity, and sensitivity, precisely evaluating disease status and serving as an effective endpoint indicator in clinical trials of idiopathic pulmonary fibrosis (IPF) [11]. The 6MWT predicts outcomes in ILD patients effectively. In a study involving newly diagnosed IPF patients [12], a 6MWD ≤72% of the predicted value was a significant independent predictor of mortality. Additionally, automated recording systems have been developed for remote assessment or recording of biomechanical gait parameters during the 6MWT [13], focusing on recording respiratory-related data, especially peripheral capillary oxygen saturation (SpO₂), which can predict long-term mortality and deterioration, enhance the benefits of home pulmonary rehabilitation, and promptly report adverse events and risks [14].

Therefore, this systematic review and meta-analysis was conducted to deepen the comprehension of how different characteristics of pulmonary rehabilitation affect their therapeutic outcomes and to test the hypothesis that pulmonary rehabilitation can enhance exercise endurance and quality of life in ILD patients.

## 2. Materials and methods

### 2.1. Protocol and guidance

Study design, literature screening, data extraction, and result reporting were all framed by the Preferred Reporting Items for Systematic Reviews and Meta-Analyses (PRISMA) guidelines to ensure methodological transparency and reporting completeness [15] (Supplementary material 1). The review protocol has been registered in the International Prospective Register of Systematic Reviews (PROSPERO; NO.CRD42025640248).

### 2.2. Information sources and eligibility criteria

A systematic and comprehensive literature search was conducted across multiple databases, including PubMed, Embase, Web of Science, Cochrane Library, and clinical trial databases etc., with the search period spanning from each database’s inception to June 2025. The aim was to evaluate the improvement in activity endurance and quality of life in patients among ILD receiving pulmonary rehabilitation programs. This study included randomized controlled trials, controlled clinical trials, controlled observational studies (prospective or retrospective), and non-controlled studies (e.g. case reports and case series). Excluded were theoretical reviews, preclinical studies, conference abstracts, and consensus reports.

The search keywords used were ‘Diffuse Parenchymal Lung Disease’ OR ‘Interstitial Lung Diseases’ OR ‘Diffuse Parenchymal Lung Diseases’ OR ‘Interstitial Lung Disease’ OR ‘Lung Disease, Interstitial’ OR ‘Pneumonia, Interstitial’ OR ‘Interstitial Pneumonia’ OR ‘Interstitial Pneumonias’ OR ‘Pneumonias, Interstitial’ OR ‘Pneumonitis, Interstitial’ OR ‘Interstitial Pneumonitides’ OR ‘Interstitial Pneumonitis’ OR ‘Pneumonitides, Interstitial’ AND ‘pulmonary rehabilitation’. Studies were screened based on research topics and types without language restrictions.

### 2.3. Study selection and data extraction

Following keyword searching, we used EndNote X9 to remove duplicates and further screened the literature. Two investigators independently excluded studies that did not meet the research objectives or standards based on the titles and abstracts. In the event of disagreements, a third investigators was consulted for final decision-making. Subsequently, the basic information of the studies, baseline characteristics of patients, specific intervention methods, and pre–post results need to be extracted, and finally presented in a table. Additionally, the Cochrane Collaboration’s risk-of-bias tool was employed to evaluate bias risk.

### 2.4. Data analysis

We used RevMan 5 for literature quality evaluation and risk of bias assessment (including plotting). Continuous variables were expressed as mean difference (MD), while dichotomous variables were presented as odds ratio (OR). The effect indices were reported with 95% confidence intervals (CI). Heterogeneity among studies was evaluated by the *I*^2^ test. A fixed-effects model was used for low heterogeneity (*I*^2^ < 50%), whereas a random-effects model was applied for high heterogeneity (*I*^2^ ≥ 50%). The alpha level for statistical significance was predefined at 0.05. Sequential leave-one-out deletion was performed for sensitivity analysis to comprehensively evaluate the relative impact of each study. Funnel plots were generated using Stata 17 for analyses with more than 10 studies to visualize publication bias. Egger’s linear regression test and Begg’s test were performed to assess publication bias; the trim-and-fill method was implemented for correction when *p* < 0.05.

## 3. Results

### 3.1. Study properties

**Figure 1.**
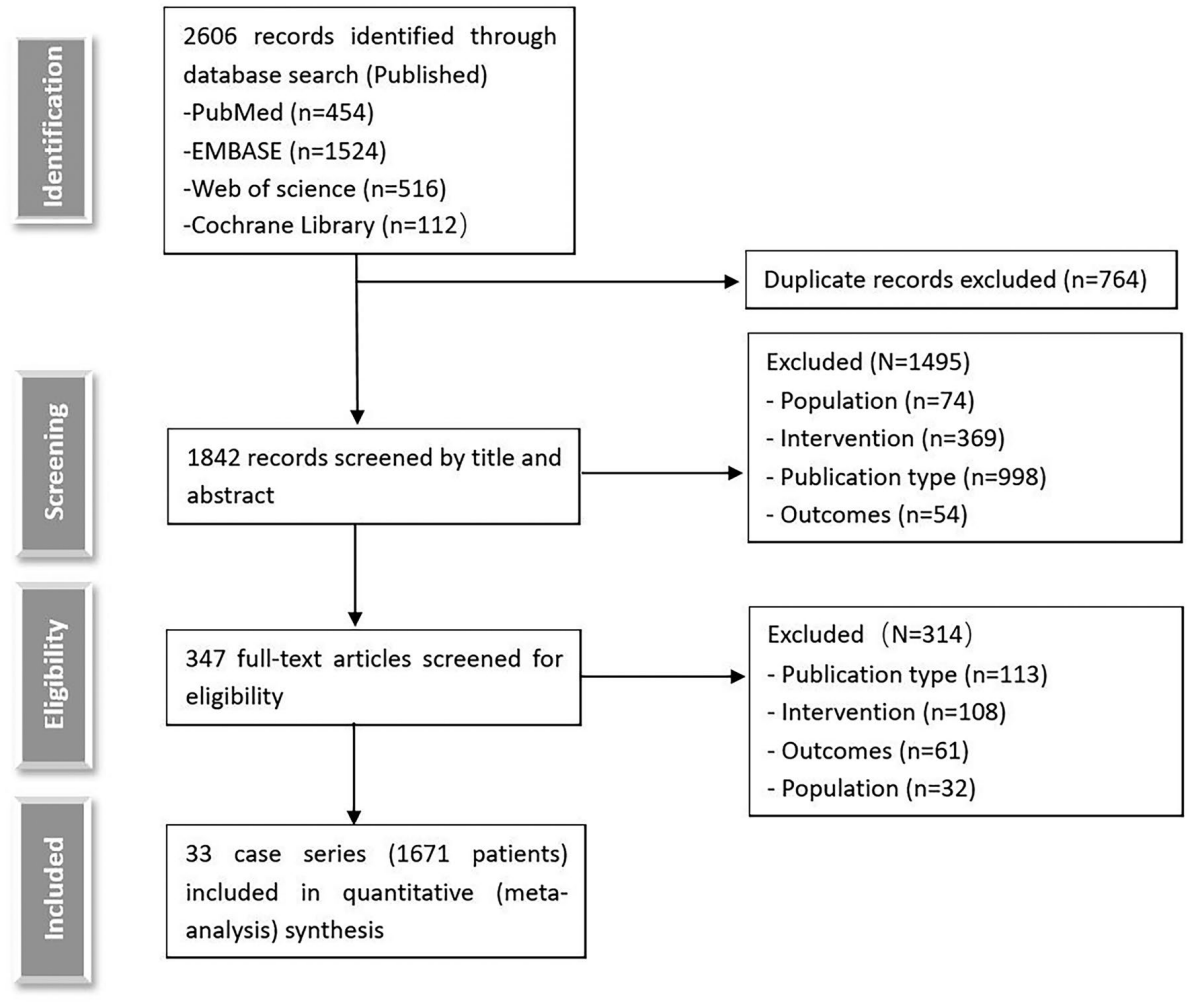
Flow diagram illustrating the procedure for study identification.

**Figure 2.**
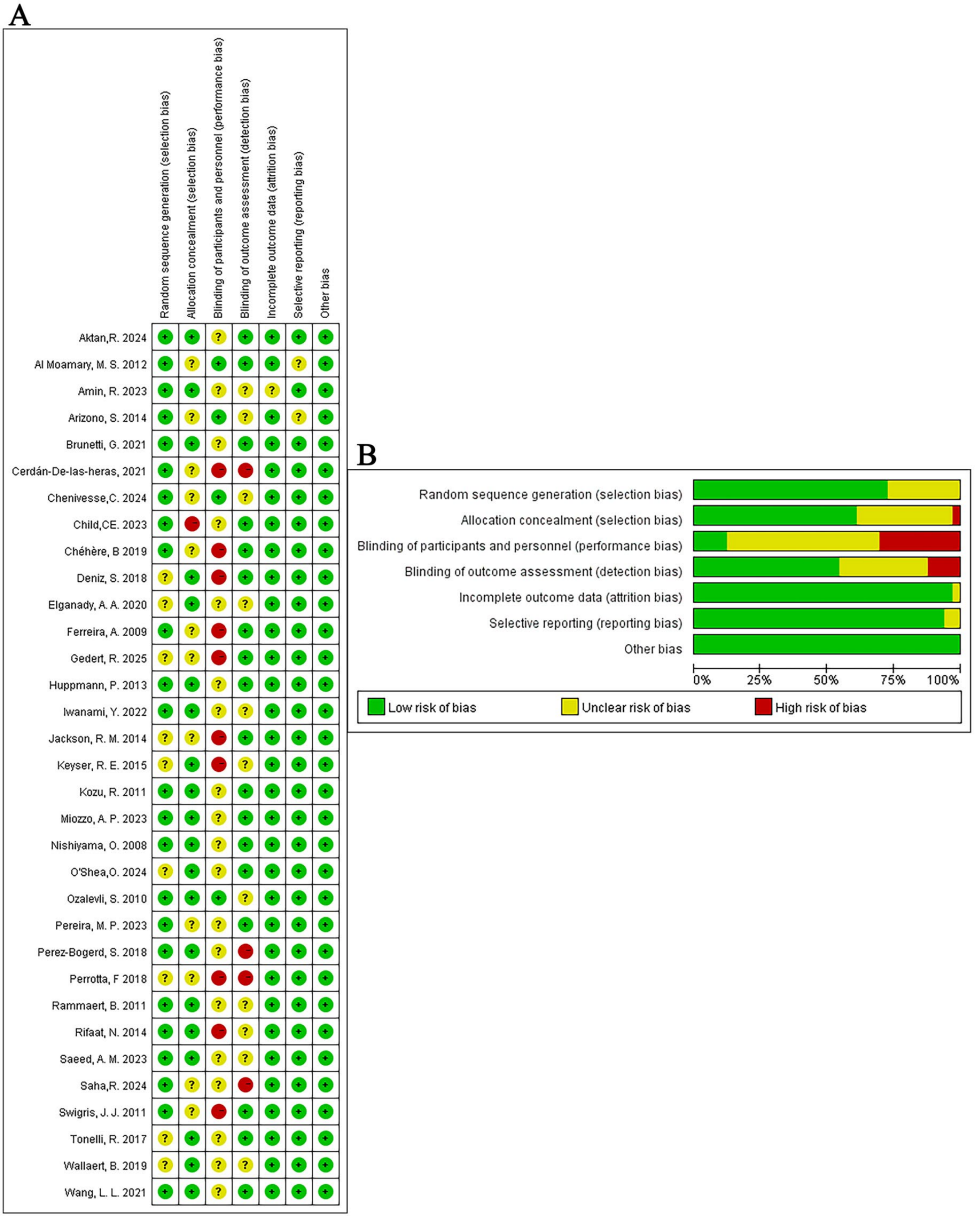
Risk of bias assessment for the randomized trials included in the meta-analysis. (A) Bias risk summary; (B) Bias risk graph. Symbols: (+) indicates low bias risk; (?) denotes unclear bias risk; (–) signifies high bias risk.

As illustrated in Figure 1, we retrieved 2606 relevant studies from four databases, excluding 764 duplicate articles. After screening the abstracts, we selected 347 articles for full-text review. We ultimately excluded 314 studies and included 33 studies for meta-analysis [16–48]. The 33 studies involved a total of 1671 ILD patients. Table 1 presents the baseline information of the included studies, and the summarized characteristics of different pulmonary rehabilitation treatments are presented in Table 2. The Cochrane Collaboration’s bias risk assessment tool (Figure 2) revealed low ratings for performance and detection bias, likely attributable to the inability to effectively blind participants, while selection bias, attrition bias, reporting bias, and other biases exhibited low risk.

**Table 1.** Characteristics of the studies included in this meta-analysis.

Authors	Year	Country	Study type	duration, m	Enrollment, n	Age, y	Male, *n*%	BMI, kg/m^2^	FEV1, % predicted	FEV1/FVC	FVC, % predicted	DLCO, % predicted
Miozzo, A. P. [16]	2023	Brazil	Retrospective	3	15	56 ± 9.13	50	26.4 ± 5.69	53 ± 17	109 ± 8	NA	50 ± 12
Pereira, M. P. [17]	2023	Brazil	Retrospective	3	16	60 ± 9	81.3	25.9 ± 1.42	55 ± 13	87 ± 8	NA	39 ± 11
Swigris, J. J. [18]	2011	America	Prospective	1.5	21	71.5 ± 7.4	85.7	NA	NA	NA	73 ± 22	38 ± 13
Perrotta, F. [19]	2018	UK	Retrospective	6	1	50	100	NA	NA	NA	NA	19.11
Iwanami, Y. [20]	2022	Japan	Prospective	3	29	73.5 ± 8.9	62	22.5 ± 3.9	95.1 ± 28.6	87.7 ± 15.7	77.9 ± 23.2	57.8 ± 18.1
Chéhère, B. [21]	2019	France	Prospective	2	19	65 ± 9	78.9	30 ± 6	73 ± 12	NA	75 ± 13	40 ± 8
Keyser, R. E. [22]	2015	America	Prospective	2.5	13	57.1 ± 9.1	38.4	28.3 ± 4.4	NA	82.4 ± 4.7	NA	39.6 ± 14.7
Kozu, R. [23]	2011	Japan	Prospective	2	45	67.5 ± 7.8	82.2	21.2 ± 3.3	80.1 ± 17.3	NA	68.6 ± 16	38 ± 82
Deniz, S. [24]	2018	Turkey	Prospective	2	57	60 ± 10	47.4	29 ± 5	68.7 ± 13.7	85.1 ± 8	NA	38.5 ± 11.9
Tonelli, R. [25]	2017	France	Prospective	0.75	41	66.9 ± 10.9	65.9	28.1 ± 3.4	78.7 ± 20.7	NA	74.5 ± 21.4	45.5 ± 20.9
Rifaat, N. [26]	2014	Egypt	Prospective	2	30	54.4 ± 6.1	26.7	NA	NA	NA	51.9 ± 13.6	62.2 ± 13.5
Saeed, A. M. [27]	2023	Egypt	Prospective	2	20	49.00 ± 8.44	30	NA	48.61 ± 7.85	78.30 ± 3.35	62.12 ± 10.01	NA
Ferreira, A. [28]	2009	America	Retrospective	2	99	66 ± 13	54.5	NA	NA	NA	62 ± 20	40 ± 14
Huppmann, P. [29]	2013	Germany	Retrospective	1	402	59.9 ± 0.6	50	26.7 ± 0.3	55 ± 1	NA	NA	NA
Nishiyama, O. [30]	2008	Japan	Prospective	2.5	13	68.1 ± 8.9	7.7	23 ± 3.8	73.3 ± 15	78.8 ± 8.2	66.1 ± 13.2	59.4 ± 16.7
Arizono, S. [31]	2014	Japan	Prospective	2.5	24	69.4 ± 7.4	66.7	NA	69.4 ± 7.4	69.4 ± 7.4	NA	49.7 ± 15.9
Jackson, R. M. [32]	2014	America	Prospective	3	11	71 ± 6	NA	NA	NA	84 ± 4	60 ± 11	44 ± 11
Rammaert, B. [33]	2011	France	Prospective	2	13	67 ± 13	62	29 ± 5	70 ± 15	NA	67 ± 14	32 ± 13
Al Moamary, M. S. [34]	2012	Saudi Arabia	Retrospective	3	21	61.0 ± 9.4	28.6	28.9 ± 8.4	60.3 ± 16.9	77.7 ± 14.7	64.4 ± 15.5	NA
Amin, R. [35]	2023	India	Prospective	1	40	60 ± 9	52.5	19.1 ± 3.04	NA	NA	76.7 ± 19.35	47 ± 9.76
Wallaert, B. [36]	2019	France	Prospective	2	112	66.8 ± 10.2	62.5	27.4 ± 4.8	70.1 ± 19.4	NA	69.7 ± 19.7	35.9 ± 12.7
Brunetti, G. [37]	2021	Italy	Retrospective	1	240	71 ± 8.73	64.17	28.51 ± 5.38	NA	NA	NA	42.63 ± 17.77
Perez-Bogerd, S. [38]	2018	Belgium	Prospective	6	30	64 ± 13	73	28 ± 4	NA	NA	NA	41 ± 13
Elganady, A. A. [39]	2020	Egypt	Prospective	1.5	20	50.25 ± 8.66	75	NA	45.55 ± 18.11	109.0 ± 18.08	43.91 ± 25.15	NA
Wang, L. L. [40]	2021	China	Prospective	3	30	55.9 ± 7.2	53.3	26.9 ± 6.3	64.3 ± 9.9	NA	72.8 ± 8.3	44.2 ± 12.3
Ozalevli, S. [41]	2010	Turkey	Prospective	3	17	62.8 ± 8.5	75	26.3 ± 3.8	64.7 ± 7.2	82.7 ± 5.6	64.7 ± 7.2	68.0 ± 32.3
Cerdán-D-l-heras, J. [42]	2021	Denmark	Prospective	3	15	70.1 ± 8.8	86.6	NA	NA	NA	76.73 ± 16.4	46.46 ± 11.0
Child, CE. [43]	2023	America	Prospective	3	15	49 ± 7.8	0	25.1 ± 4.5	66.8 ± 20.1	65.4 ± 14.1	81.6 ± 18.5	NA
O’Shea, O. [44]	2024	Ireland	Prospective	2.5	16	71.4 ± 11.5	43.75	NA	92.1 ± 18.9	80 ± 0	85.2 ± 19.2	57.4 ± 20.1
Aktan, R. [45]	2024	Turkey	Prospective	2	14	66.5 ± 5.5	57.1	26.5 ± 5.5	89.8 ± 17.4	NA	84.6 ± 16.9	60.2 ± 34.6
Chenivesse, C. [46]	2024	France	Retrospective	2	166	68.6 ± 10.7	65.06	29 ± 8.6	66.4 ± 19.2	NA	69.6 ± 20.8	35.8 ± 14.0
Saha, R. [47]	2024	India	Prospective	2	45	62.0 ± 11.0	55.6	NA	45.86 ± 4.35	84 ± 5	42.88 ± 4.70	25.35 ± 4.86
Gedert, R. [48]	2025	America	Prospective	2	21	65.5 ± 8	38.09	NA	NA	NA	64.8 ± 20.4	45.3 ± 12.6

FEV1: forced expiratory volume in one second; FVC: forced vital capacity; DLCO: diffusing capacity of the lungs for carbon monoxide; m: months; y: years; n: number; NA: not available.

**Table 2.** Characteristics of different pulmonary rehabilitation treatments of the studies included in this meta-analysis.

Authors	Training duration, w	Training mode	Training content
Miozzo, A. P. [16]	12 weeks	Hospital	Endurance (treadmill), strengthening (upper and lower limbs)
Pereira, M. P. [17]	12 weeks	Hospital	Endurance (treadmill), strengthening (upper and lower limbs)
Swigris, J. J. [18]	6–8 weeks	Hospital	Endurance (treadmill, bikes), strengthening, breathing exercises
Perrotta, F. [19]	8 weeks	Hospital	Endurance (treadmill), strengthening (upper and lower limbs)
Iwanami, Y. [20]	12 weeks	Hospital	Endurance (treadmill), strengthening (upper and lower limbs), breathing exercises
Chéhère, B. [21]	8 weeks	Hospital	Endurance (cycling), strengthening (upper and lower limbs)
Keyser, R. E. [22]	10 weeks	NA	Endurance (walking)
Kozu, R. [23]	8 weeks	Hospital and home-based	Endurance (cycling), strengthening (upper and lower limbs), breathing exercises
Eniz, S. [24]	8 weeks	NA	Endurance (treadmill), strengthening (upper and lower limbs), breathing exercises
Tonelli, R. [25]	4 weeks	Hospital	Endurance (treadmill, bikes), strengthening, breathing exercises
Rifaat, N. [26]	8 weeks	Hospital	Endurance (cycling), strengthening (upper and lower limbs), breathing exercises
Ferreira, A. [27]	6–8 weeks	Hospital	Endurance, strengthening, breathing exercises
Huppmann, P. [28]	4 weeks	Hospital	Endurance (treadmill, bikes), strengthening, breathing exercises
Nishiyama, O. [29]	10 weeks	Hospital	Endurance (treadmill), strengthening (upper and lower limbs)
Arizono, S. [30]	10 weeks	Hospital	Endurance (cycling), strengthening (upper and lower limbs), breathing exercises
Jackson, R. M. [31]	12 weeks	NA	Endurance (treadmill, cycling), strengthening
Rammaert, B. [32]	8 weeks	Home-based	Endurance (cycling), strengthening
Al Moamary, M. S. [33]	8–12 weeks	Hospital	Endurance, strengthening (upper and lower limbs)
Amin, R. [34]	4 weeks	Hospital and home-based	Endurance, strengthening (upper and lower limbs)
Wallaert, B. [35]	8 weeks	Home-based	Endurance (cycling), strengthening
Brunetti, G. [36]	3–4 weeks	Hospital	Endurance (cycling), strengthening (upper and lower limbs)
Perez-Bogerd, S. [37]	24 weeks	Hospital	Endurance, strengthening
Elganady, A. A. [38]	6 weeks	NA	Endurance (treadmill), strengthening (upper and lower limbs), breathing exercises
Saeed, A. [39]	8 weeks	Hospital and home-based	Endurance (walking), strengthening (upper limbs), breathing exercises
Wang, L. L. [40]	12 weeks	Home-based	Endurance, strengthening, breathing exercises
Ozalevli, S. [41]	12 weeks	Home-based	Endurance (walking), breathing exercises
Cerdán-De-Las-Heras, J. [42]	12 weeks	Tele-rehabilitation	Virtual autonomous physiotherapist agent (VAPA)
Child, CE. [43]	12 weeks	Tele-rehabilitation	Remote monitoring-enabled exercise prescription
O’Shea, O. [44]	10 weeks	Tele-rehabilitation	Virtual pulmonary rehabilitation (VPR)
Aktan, R. [45]	8 weeks	Tele-rehabilitation	Tele-rehabilitation-assisted inspiratory muscle training
Chenivesse, C. [46]	8 weeks	Home-based	Endurance (cycling), strengthening
Saha, R. [47]	8 weeks	Home-based	Endurance (walking, jogging), strengthening (upper and lower limbs), breathing exercises
Gedert, R. [48]	8 weeks	Hospital	Endurance (cycling, walking), strengthening (upper and lower limbs)

### 3.2. Meta-analysis outcome

#### 3.2.1. 6-Min walk distance (6MWD)

**Figure 3.**
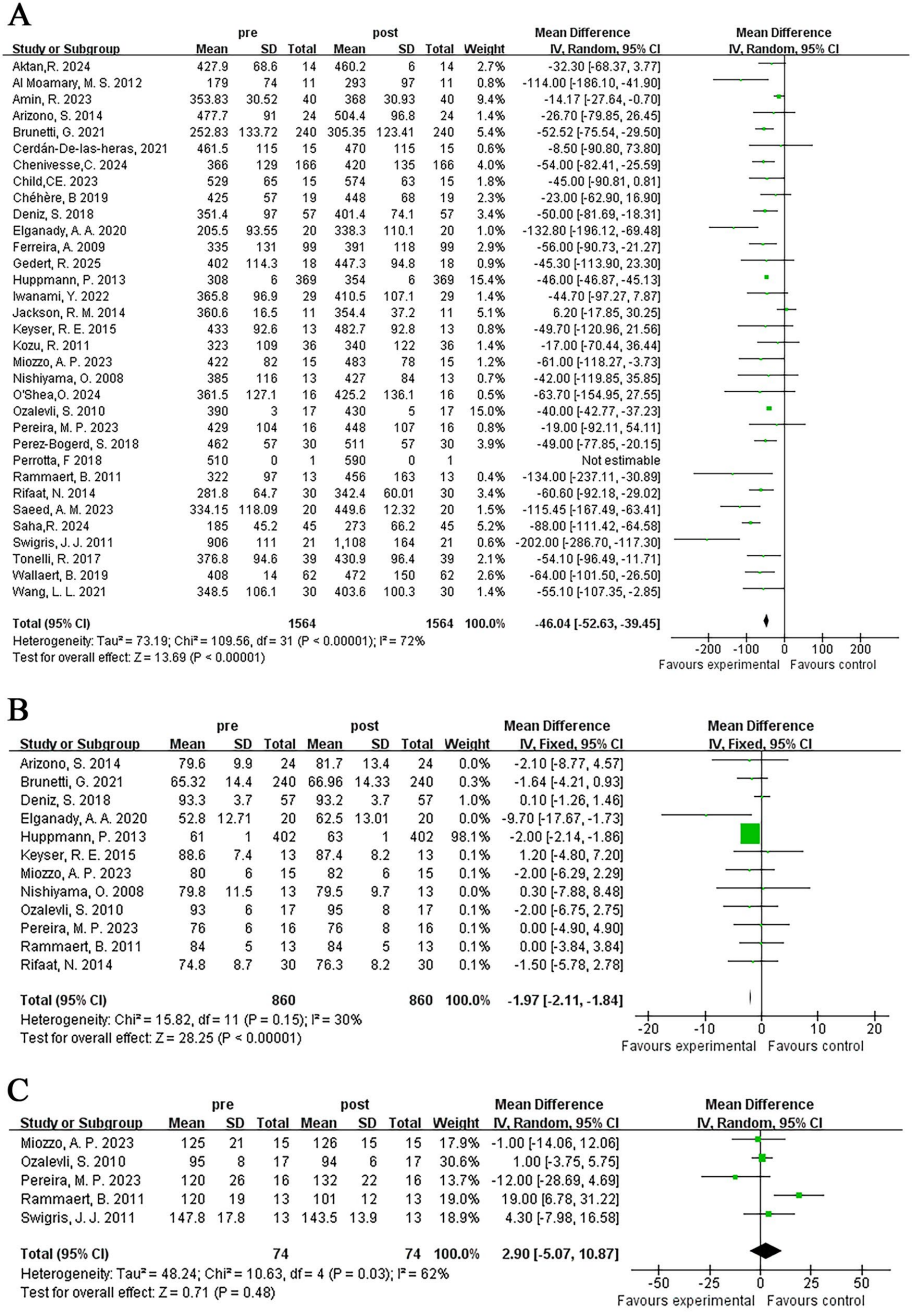
The effect of pulmonary rehabilitation on the exercise endurance in ILD patients; (A) 6WMD; (B) lowest oxygen saturation during 6MWT; (C) peak heart rate during 6MWT.

The variation in 6MWD was adopted to assess alterations in exercise endurance among ILD patients after pulmonary rehabilitation of different durations and methods in 33 studies. Heterogeneity testing indicated heterogeneity (*I*^2^ = 72%), and therefore a random-effects model was used for meta-analysis. The results showed a significant increase in 6MWD after pulmonary rehabilitation, with statistical significance [MD = −46.04; 95% CI (−52.63, −39.45); *p* < 0.001] (Figure 3A).

#### 3.2.2. Lowest oxygen saturation during 6MWT

Twelve studies reported the lowest oxygen saturation recorded during the 6MWT in ILD patients, involving a total of 860 patients. Heterogeneity testing indicated homogeneity (*I*^2^ = 30%). Thus, a fixed-effects model was applied in the meta-analysis. It was observed the lowest oxygen saturation during exercise improved significantly after pulmonary rehabilitation, with statistical significance [MD = −1.97; 95% CI (−2.11, −1.84); *p* < 0.001] (Figure 3B).

#### 3.2.3. Peak heart rate during 6MWT

Five studies reported peak heart rate changes during the 6MWT in ILD patients, involving a total of 74 patients. Significant heterogeneity (*I*^2^ = 62%) was identified, leading to the adoption of a random effects model for meta-analysis. The findings revealed that peak heart rate changes failed to achieve statistical significance [MD = 2.9; 95% CI (−5.07, 10.87); *p* = 0.48] (Figure 3C).

#### 3.2.4. Borg scale score

**Figure 4.**
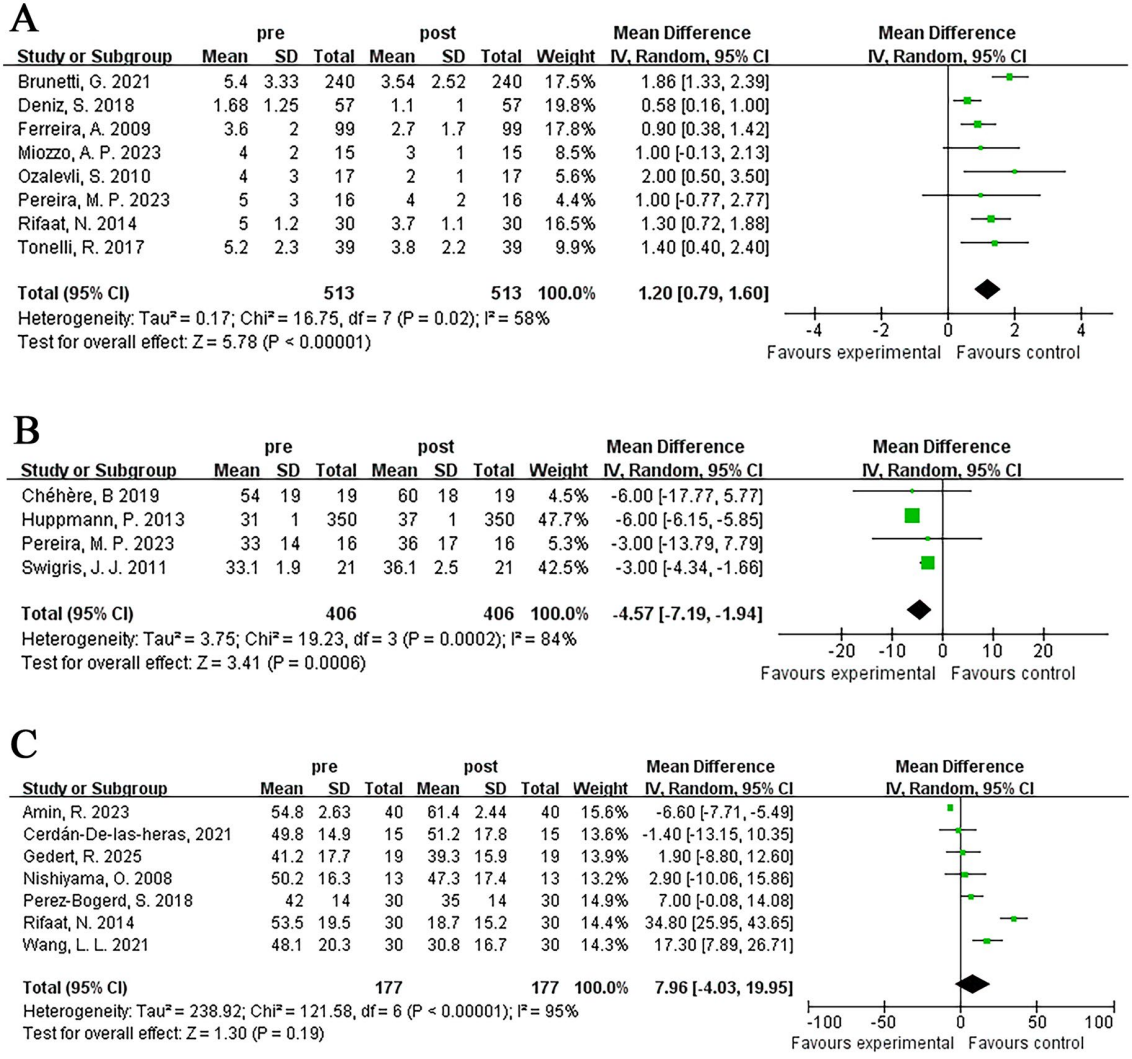
The impact of pulmonary rehabilitation on quality of life among ILD patients; (A) borg scale score; (B) SF-36; (C) SGRQ.

Eight studies reported borg scale scores pre- and post-pulmonary rehabilitation, involving a total of 513 patients. Heterogeneity testing indicated heterogeneity (*I*^2^ = 58%), and consequently a random-effects model was employed. The results demonstrated significant differences [MD = 1.20; 95% CI (0.79, 1.60); *p* < 0.001] (Figure 4A).

#### 3.2.5. The MOS 36-Item Short Form Health Survey (SF-36)

Four studies reported changes in SF-36 scores pre- and post-pulmonary rehabilitation, encompassing a total of 406 patients. Heterogeneity testing indicated heterogeneity (*I*^2^ = 84%), thus, a random-effects model was applied in the meta-analysis. The findings indicated significant differences [MD = −4.57; 95% CI (−7.19, −1.94); *p* < 0.001] (Figure 4B).

#### 3.2.6. St. George’s respiratory questionnaire (SGRQ)

Seven studies (177 patients) included SGRQ scores pre- and post-pulmonary rehabilitation. Heterogeneity testing indicated heterogeneity (*I*^2^ = 95%), and a random-effects model was adopted. The results revealed no significant difference, likely attributed to the substantial heterogeneity among included studies [MD = 7.96; 95% CI (−4.03, 19.95); *p* = 0.19] (Figure 4C).

### 3.3. Subgroup analysis

#### 3.3.1. Duration of pulmonary rehabilitation programs

Based on the duration of pulmonary rehabilitation training for ILD patients, we grouped them into ≤4 weeks, >4 weeks to ≤8 weeks, and >8 weeks to ≤12 weeks. Subgroup heterogeneity testing indicated heterogeneity (*I*^2^ = 73%), thus random-effects model was adopted. The results of the three subgroups all indicated that pulmonary rehabilitation programs of different durations could increase 6MWD. Interestingly, the duration of >4 weeks to ≤8 weeks showed the most significant improvement in exercise endurance [MD = −66.87; 95% CI (−85.40, −48.35); *p* < 0.001], while the results in the ≤4 weeks training group [MD = −39.28; 95% CI (−59.33, −19.22); *p* < 0.001] and the >8 weeks to ≤12 weeks training group [MD = −36.75; 95% CI (−52.66, −20.85); *p* < 0.001] were similar, with statistically significant differences between subgroups (*p* = 0.04) (Figure 5A).

Figure 5.Subgroup analysis of the effects of different characteristics of pulmonary rehabilitation on 6MWD in ILD; (A) Duration; (B) Training content; (C) Implementation methods.

**Figure:**
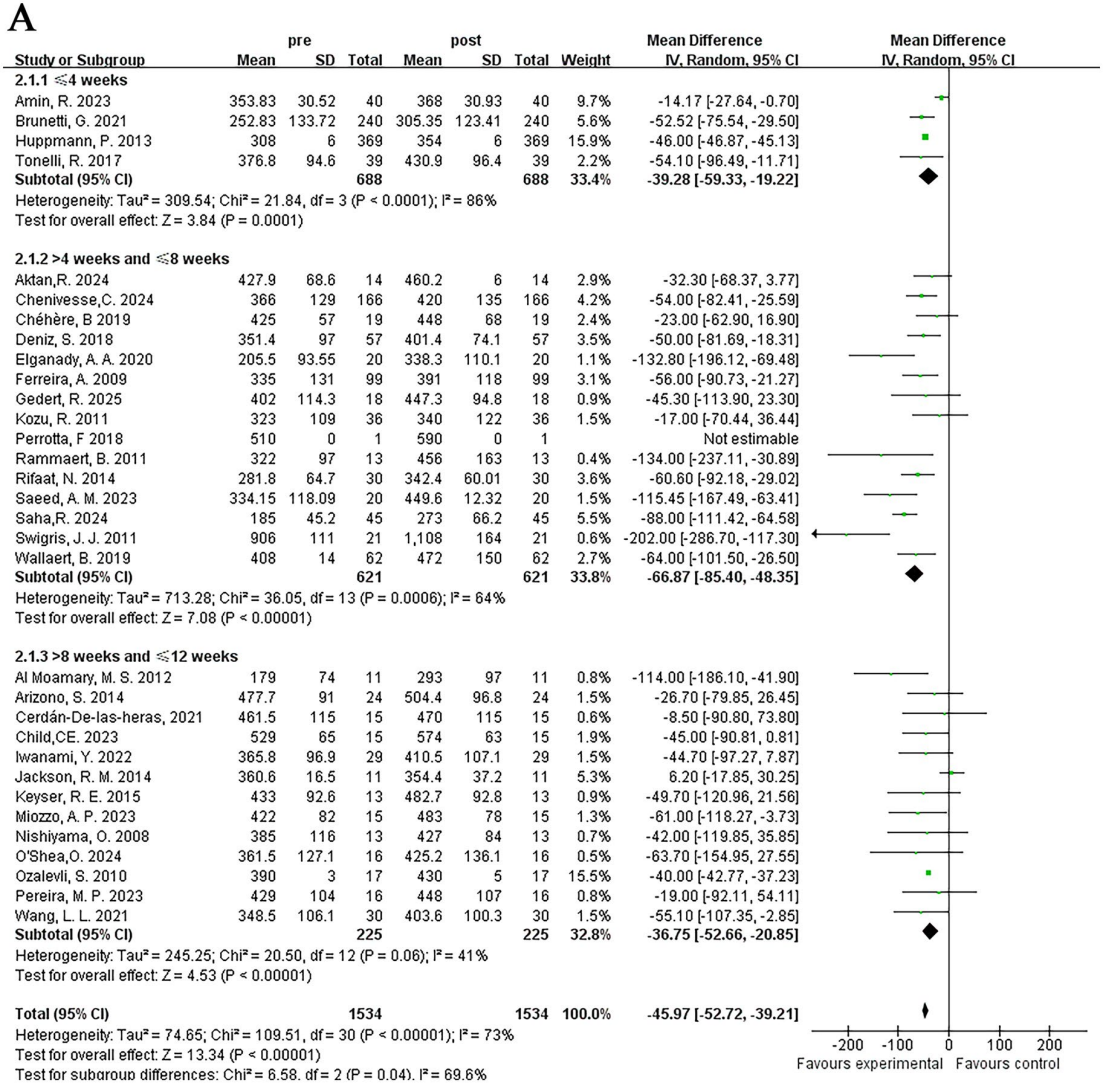

**Figure:**
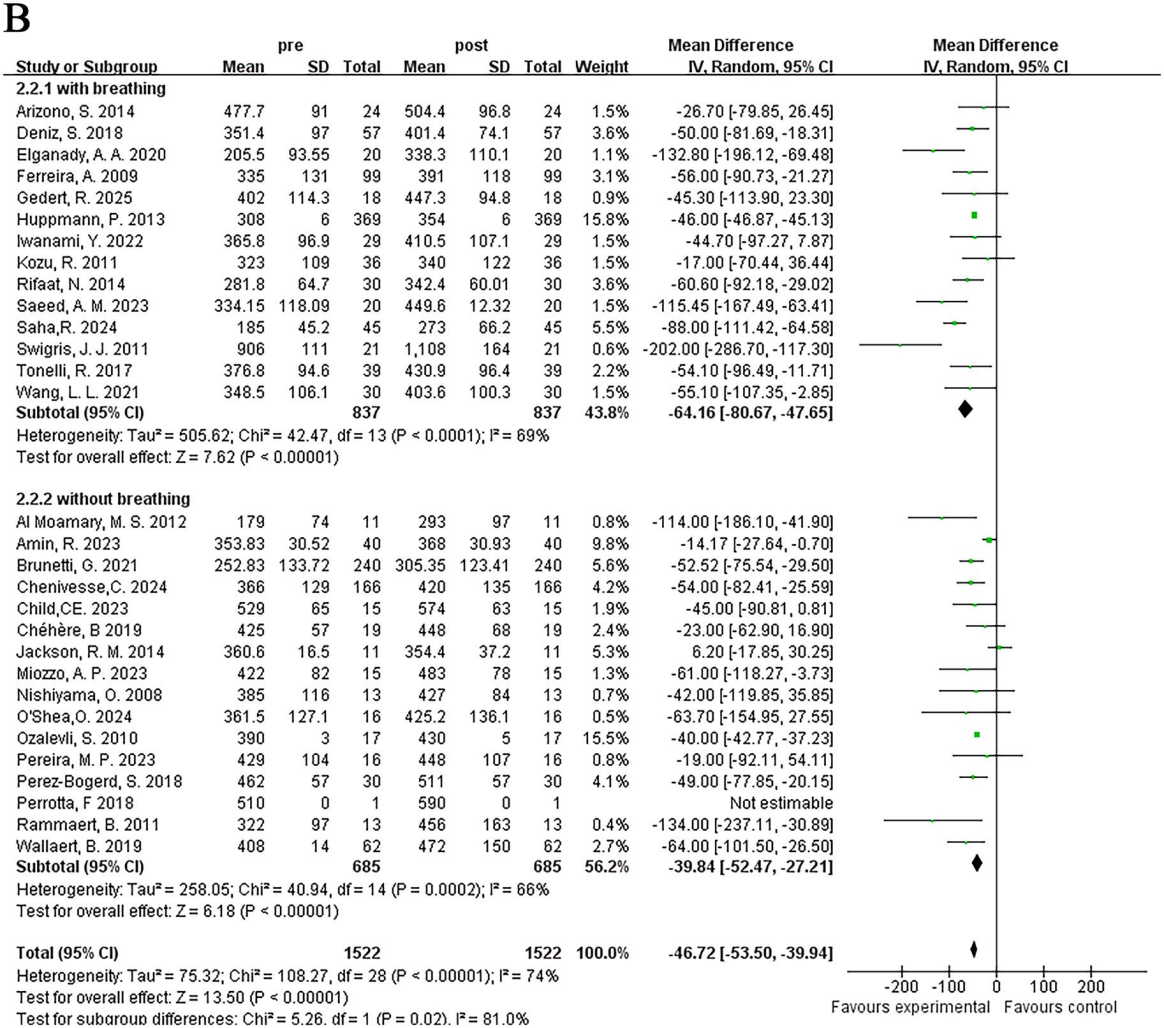

**Figure:**
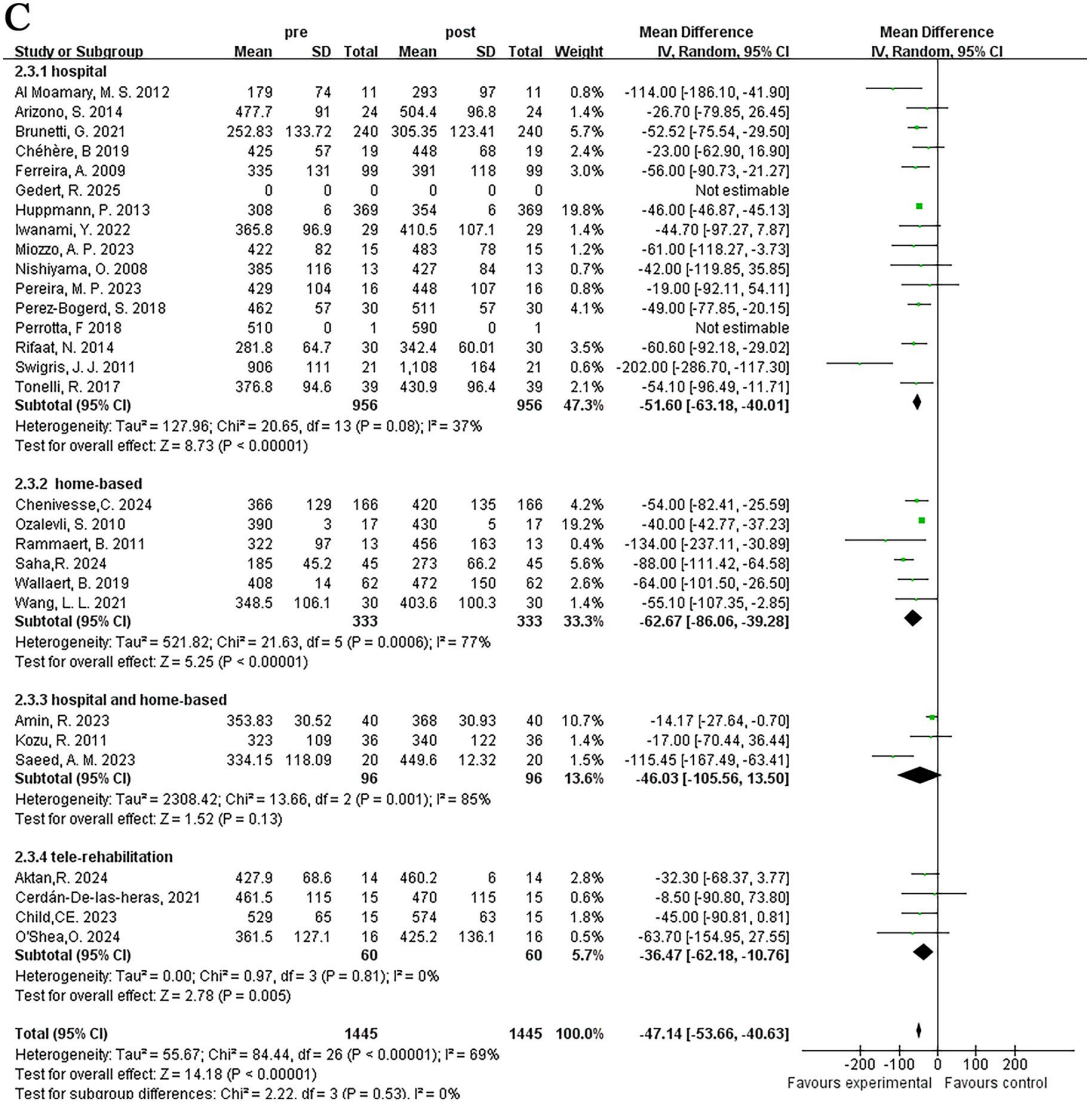

#### 3.3.2. Training content of pulmonary rehabilitation programs

Commonly applied pulmonary rehabilitation programs consist of endurance training, strength training, and breathing training. Among the 33 studies included in this analysis, 14 studies included all three types of training, 16 studies included endurance and strength training, 1 study included endurance and breathing training, 1 study only included endurance training, and 1 study had unclear description. To reduce heterogeneity and clarify the function of breathing training in pulmonary rehabilitation, subgroup analysis was conducted based on whether breathing training was included in addition to endurance and strength training. Subgroup heterogeneity testing indicated heterogeneity (*I*^2^ = 74%), thus a random-effects model was applied in the meta-analysis. The results of the two subgroups indicated that pulmonary rehabilitation programs, whether or not they included breathing training, significantly improved 6MWD. The endurance + strength + breathing training group [MD = −64.16; 95% CI (−80.67, −47.65); *p* < 0.001] showed greater improvement in exercise endurance than the endurance + strength training group [MD = −39.84; 95% CI (−52.47, −27.21); *p* < 0.001], with statistically significant differences between groups (*p* = 0.02) (Figure 5B).

#### 3.3.3. Implementation methods of pulmonary rehabilitation programs

Additionally, studies that clearly described the specific implementation methods of pulmonary rehabilitation were included, and grouped according to different methods. The 33 studies were divided into medical guidance group, home rehabilitation group, medical guidance combined with home rehabilitation group, and new remote assistance group, excluding 4 studies with undefined methods. Subgroup analysis was conducted accordingly, with subgroup heterogeneity testing indicating heterogeneity (*I*^2^ = 69%), a random-effects model was used. The medical guidance group [MD = −51.60; 95% CI (−63.18, −40.01); *p* < 0.001], the home rehabilitation group [MD = −62.67; 95% CI (−86.06, −39.28); *p* < 0.001], and the new remote assistance group [MD = −36.47; 95% CI (−62.18, 10.76); *p* = 0.005] could all improve patients’ exercise endurance, yet the improvement degree of the new remote assistance group was relatively weaker. It was observed in the study that the medical guidance combined with home rehabilitation group [MD = −46.03; 95% CI (−105.56, −13.50); *p* = 0.13] had no significant improvement in exercise endurance. No statistically significant differences were observed between subgroups (*p* = 0.53) (Figure 5C).

### 3.4. Publication bias

**Figure 6.**
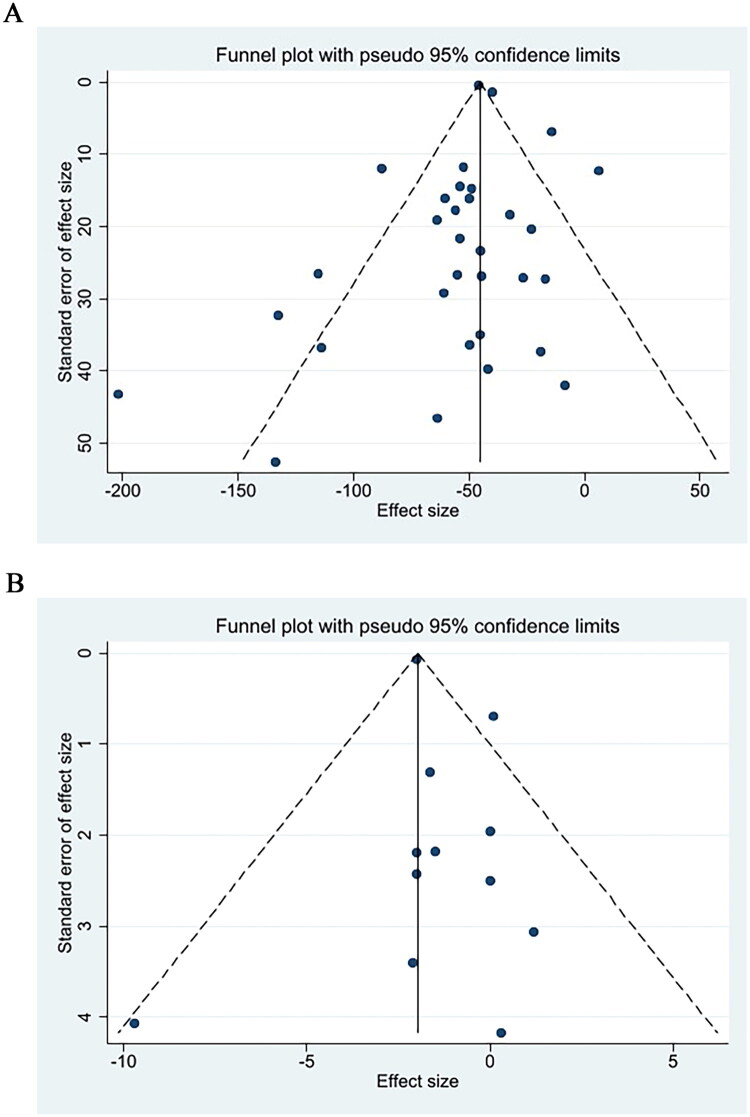
Funnel plot for evaluating publication bias. Each data point denotes an independent study for the specified association. (A) 6MWT; (B) lowest oxygen saturation during 6MWT.

Publication bias was quantitatively evaluated by Egger’s linear regression test and Begg’s rank correlation test. Egger’s test (*t* = 0.55, *p* = 0.590) and Begg’s test (*z* = 0.18, *p* = 0.858) revealed no evidence of publication bias for 6MWD (Figure 6A). Similarly, no publication bias was detected for the lowest oxygen saturation during the 6MWT, as indicated by Begg’s test (*z* = 0.48, *p* = 0.631) and Egger’s test (*t* = 1.20, *p* = 0.257) (Figure 6B).

## 4. Discussion

For patients with ILD, it is recommended to comprehensive treatment combining pulmonary rehabilitation as early as possible under antifibrotic and immunomodulatory drug therapy. A systematic review and meta-analysis was conducted to evaluate the efficacy of standardized pulmonary rehabilitation in enhancing exercise tolerance and quality of life in patients with ILD.

Studies have confirmed [49–52] that the 6MWD plays an important role in assessing severity, providing feedback on treatment effects, and predicting morbidity and mortality risks. The advantages of pulmonary rehabilitation in delaying disease progression and reducing mortality risk can be evaluated through long-term follow-up, while assessing the clinical utility of 6MWD as a prognostic marker for ILD patients.

Progressive hypoxemia is a characteristic of ILD, and exertional hypoxemia [53] is defined as the lowest SpO_2_ < 88% during the 6MWT in room air. This study verified pulmonary rehabilitation can significantly reduce the occurrence and progression of exertional hypoxemia. Therefore, early identification of ILD patients with clinically significant hypoxemia and pulmonary rehabilitation treatment are crucial to provide symptomatic benefits and potentially improve survival. The peak heart rate changes collected during the 6MWT did not show significant differences before and after rehabilitation, possibly related to differences in cardiac function. However, studies have shown that heart rate changes during the 6MWT are a standalone predictor of the patients with pulmonary hypertension [54], and further evaluation of heart rate changes in ILD patients after removing confounding factors is needed.

Health-related quality of life (HRQoL) is a multidimensional concept integrating key health domains such as physical, psychological, and social well-being [55]; the SF-36 serves as the most commonly used standardized tool to capture this complex construct [56]. This study also verified via the SF-36 that pulmonary rehabilitation can significantly improves the quality of life across domains such as physical function and psychological well-being. Similarly, the Borg scale score also demonstrated that pulmonary rehabilitation can reduces subjective fatigue during exercise and enhances exercise endurance.

The IPF guidelines recommend pulmonary rehabilitation as a major component of non-pharmacological treatment; evidence of long-term effects remains controversial [57,58]. This meta-analysis, which included all ILD patients undergoing pulmonary rehabilitation, showed that subgroup analysis based on rehabilitation duration indicated that the 4–8-week group had the best improvement in exercise endurance. ILD patients exhibit parenchymal lung injury and progressive fibrosis as primary pathological changes, leading to gas exchange dysfunction. This manifests as prominent dyspnea, reduced exercise tolerance, and markedly impaired quality of life [59]. Studies have demonstrated that quadriceps weakness and sedentary behavior exist in IPF. Moreover, studies have shown that high mortality is associated with impaired exercise tolerance, decreased activity levels, and excessively low fat-free mass index [60]. The use of corticosteroids and immunosuppressants can also lead to muscle atrophy and other negative effects [61], so longer rehabilitation training is needed to induce muscular adaptations and improve their exercise capacity. Training periods shorter than 4 weeks may not yield significant improvements. Our study results indicate that the benefits of long-term pulmonary rehabilitation diminish, possibly due to fatigue from long-term high-intensity exercise leading to decreased immunity or secondary infections in suboptimal rehabilitation environments, which exacerbate dyspnea. High-intensity exercise training increases the burden on the heart and blood pressure fluctuations, increasing the risk of serious complications such as cerebrovascular accidents. Attention should also be paid to patients’ mental health to avoid anxiety and depression due to high expectations, which can dampen enthusiasm for rehabilitation and form a vicious cycle [38,62]. To maximize the long-term benefits for ILD patients and explore the optimal maintenance training**–**interval pattern, more multi-center large-sample studies are required in the future.

Growing evidence suggest that respiratory muscle dysfunction may well explain the refractory hypoxemia and reduced exercise tolerance in patients with ILD [63–65]. Zaki et al. [66] showed that, for ILD patients undergoing inspiratory muscle training, inspiratory muscle training combined with pulmonary rehabilitation might be more beneficial than pulmonary rehabilitation alone. Our study also indicated that endurance, resistance training combined with respiratory training is more favorable for improving exercise endurance in ILD patients (MD 64.16:46.72). For instance, pursed-lip breathing and diaphragmatic breathing can increase resistance during exhalation, enhance the strength of respiratory muscles such as the intercostal muscles and diaphragm, improve ventilatory function, facilitate gas exchange, and alleviate dyspnea. The favorable effects of breathing training can be triggered through declines in the neural respiratory drive and refinements in breathing patterns. Such alterations can correct prior mismatches between the loading and capacity of respiratory muscles [67]. Altering the original shallow and rapid breathing pattern [68] can reduce the work of respiratory muscles, enhance patients’ control over their breathing, and mitigate the aggravating effect of anxiety on dyspnea. Additionally, improved exercise endurance can boost aerobic capacity, thereby reducing ventilatory load during exercise [69].

Although ample evidence demonstrates the significant effectiveness of pulmonary rehabilitation, and relevant guidelines have provided clear recommendations, its widespread implementation has not been achieved in clinical practice [70]. The factors contributing to its low participation and completion rates are complex, with inconvenient scheduling of rehabilitation programs and the long geographical distance from pulmonary rehabilitation centers recognized as two major obstacles [71,72]. Traditionally, pulmonary rehabilitation services have been predominantly delivered in hospital centers (center-based PR) [73]. However, numerous new rehabilitation models have pioneered brand-new development directions, such as home-based pulmonary rehabilitation (home-PR) and remote pulmonary rehabilitation, with promising prospects. Numerous randomized controlled trials [74,75] and meta-analyses [76] on pulmonary rehabilitation for chronic airway diseases have shown that home-PR demonstrates equivalent efficacy to traditional center-based PR. This study’s findings demonstrate that pulmonary rehabilitation for ILD patients significantly improves exercise endurance in three settings: medical centers, home-based environments, and new remote assistance models. Among these, home rehabilitation demonstrates the most pronounced improvement in 6MWD, potentially attributed to the stable home environment and robust implementation guarantee of interventions. The remote assistance group showed relatively poor improvement efficacy, which may be related to its characteristics: remote intervention relies on equipment and networks. If the technology is immature, it will lead to a decrease in patients’ actual participation, poor continuity and integrity, and this model has relatively high requirements for patient adaptability. Notwithstanding, many current studies have proven the value of remote rehabilitation [77–79]. Notably, medical guidance combined with home rehabilitation model failed to show significant improvements in exercise capacity, likely due to high heterogeneity across included studies. Further clinical investigations with standardized design protocols and optimized data quality are needed to enhance the evidence base. Unlike other respiratory diseases, ILD leads to more severe hypoxemia [80], necessitating meticulous monitoring of blood pressure, oxygen saturation, and pulse during rehabilitation exercises to avoid intense aerobic activities that elevate intrathoracic pressure. Rehabilitation protocols must be formulated with greater caution to ensure patient safety [81]. During home or remote rehabilitation, close monitoring of vital signs and symptom fluctuations is essential to prevent adverse events. In conclusion, the safety and long-term efficacy of home and new remote rehabilitation models need to be thoroughly evaluated.

The principal limitation is the included pulmonary rehabilitation projects did not clearly classify ILD patients by etiology and pathology, making it unfeasible to validate the efficacy of pulmonary rehabilitation for specific ILD types. Similarly, we did not assess the impact of pulmonary rehabilitation on ILD patients after staging their disease progression based on lung function. Furthermore, although we synthesized results of a series of trials and performed subgroup analyses under different conditions to mitigate heterogeneity, the influence of the minimal clinically important difference (MCID) for each indicator may not have been accurately reflected. In conclusion, our ultimate goal is to determine the optimal pulmonary rehabilitation programs and plans for different types of ILD patients, which requires further confirmation through large-sample, well-designed studies.

## 5. Conclusion

Substantial evidence demonstrates that pulmonary rehabilitation is a critical therapeutic intervention for ILD patients. It significantly enhances exercise endurance and quality of life, exerting a profound positive impact on their clinical outcomes. To achieve optimal therapeutic efficacy, however, thorough consideration of patient-specific conditions and adherence to individualization principles are essential in designing pulmonary rehabilitation programs that maximize tolerance and therapeutic benefits.

## Supplementary Material

Supplementary material 1 PRISMA_2020_checklist_Revision1.docx

## Data Availability

The raw data are available from the corresponding author, Liang Dong, upon reasonable request. Interested parties may contact Dr. Liang Dong via email at dl5506@126.com to access the data.
